# Systems patientomics: the virtual *in-silico* patient

**DOI:** 10.1186/1878-5085-5-S1-A53

**Published:** 2014-02-11

**Authors:** Dimiter V Dimitrov

**Affiliations:** 1 Diavita Ltd., Varna, Bulgaria

## 

The integration of molecular sciences and computational sciences is moving the world towards a new generation of life science where physiological information from the human can be quantitatively described *in silico* via biocomputing. Systems Biology & Systems Medicine represent those emerging sciences. The development of the “Virtual Patient Avatar” will change conventional medicine into "predictive medicine" that will have the capacity to develop solutions based upon understanding of the dynamic mechanisms and the quantitative logic of human physiology. Drug discovery and clinical trials *in silico* will improve the development of products with higher efficiency and safety while reducing cost. Thus strategy must occur in the European Union to bridge the gap between academic and industry in the following ways:

• Validate and publish medical informatics data and technology models to accepted standards in order to facilitate adoption,

• Transform electronic health records to make them more accessible and interoperable,

• Encourage information sharing, engage regulatory agencies, and

• Encourage increasing financial support to grow and develop translational biomedical informatics.

